# An Antivector Vaccine Protects against a Lethal Vector-Borne Pathogen

**DOI:** 10.1371/journal.ppat.0020027

**Published:** 2006-04-07

**Authors:** Milan Labuda, Adama R Trimnell, Martina Ličková, Mária Kazimírová, Gillian M Davies, Olga Lissina, Rosie S Hails, Patricia A Nuttall

**Affiliations:** 1 Institute of Zoology, Slovak Academy of Sciences, Bratislava, Slovakia; 2 Natural Environment Research Council Centre for Ecology and Hydrology, Oxford, United Kingdom; 3 Institute of Virology, Slovak Academy of Sciences, Bratislava, Slovakia; Scripps Research Institute, United States of America

## Abstract

Vaccines that target blood-feeding disease vectors, such as mosquitoes and ticks, have the potential to protect against the many diseases caused by vector-borne pathogens. We tested the ability of an anti-tick vaccine derived from a tick cement protein (64TRP) of Rhipicephalus appendiculatus to protect mice against tick-borne encephalitis virus (TBEV) transmitted by infected Ixodes ricinus ticks. The vaccine has a “dual action” in immunized animals: when infested with ticks, the inflammatory and immune responses first disrupt the skin feeding site, resulting in impaired blood feeding, and then specific anti-64TRP antibodies cross-react with midgut antigenic epitopes, causing rupture of the tick midgut and death of engorged ticks. Three parameters were measured: “transmission,” number of uninfected nymphal ticks that became infected when cofeeding with an infected adult female tick; “support,” number of mice supporting virus transmission from the infected tick to cofeeding uninfected nymphs; and “survival,” number of mice that survived infection by tick bite and subsequent challenge by intraperitoneal inoculation of a lethal dose of TBEV. We show that one dose of the 64TRP vaccine protects mice against lethal challenge by infected ticks; control animals developed a fatal viral encephalitis. The protective effect of the 64TRP vaccine was comparable to that of a single dose of a commercial TBEV vaccine, while the transmission-blocking effect of 64TRP was better than that of the antiviral vaccine in reducing the number of animals supporting virus transmission. By contrast, the commercial antitick vaccine (TickGARD) that targets only the tick's midgut showed transmission-blocking activity but was not protective. The 64TRP vaccine demonstrates the potential to control vector-borne disease by interfering with pathogen transmission, apparently by mediating a local cutaneous inflammatory immune response at the tick-feeding site.

## Synopsis

Blood-sucking vectors such as mosquitoes and ticks transmit hundreds of micro-organisms that cause diseases like malaria and Lyme disease. Controlling so many diseases is an enormous challenge. A new idea is to make vaccines against the vectors rather than against all the individual disease agents they carry. The authors examined this hypothesis using a vaccine prepared from tick cement. This cement is secreted by ticks to help them attach to a human or animal to feed. A mouse model was used in which mice were infested with ticks infected with tick-borne encephalitis virus (TBEV), the most important vector-borne virus in Europe and northern Asia. The control mice developed fatal encephalitis and died about a week after being bitten by the infected tick. By contrast, the tick cement vaccine gave protection similar to the level seen in mice immunized with a single shot of the commercial TBEV vaccine for humans. However, a commercial tick vaccine used to control cattle ticks did not protect the mice. The authors' tick cement vaccine appeared to work by causing a cellular immune response in the skin where ticks were feeding. These results show that it is feasible to produce a vaccine against a tick that protects against the disease agent it transmits.

## Introduction

Blood-feeding parasites act as vectors of an enormous range of pathogens that cause diseases in humans and other animals. For example, a single tick species *(Ixodes ricinus)* can transmit viruses, bacteria, and protozoa that cause tick-borne encephalitis, Lyme disease, and babesiosis. Protection by immunization requires several different antipathogen vaccines, while vector control generally relies on the use of repellents, or on large-scale, repeated applications of pesticides that raise issues concerning pesticide resistance, food residues, health risks, and environmental pollution. New strategies are required to control both the vectors and pathogens they transmit.

A novel approach is antivector vaccines designed to target the vector in such a way that they protect against pathogens transmitted by the vector. Several observations suggest this may be feasible. Reduced transmission capacity of ticks fed on tick-immune animals [1–5] and humans [6] has been reported for several tick-borne pathogens, although not all [7]. For example, people who express an immune reaction against the vector tick Ixodes scapularis appear to acquire Lyme disease less frequently than those who experience no such immune response [6]. For insect vectors, bites of uninfected sand flies provide protection of mice against cutaneous leishmaniasis [8], and seroconversion of humans against sandfly vectors correlates with development of protective immunity to leishmaniasis [9]. Experimentally, it has been shown that antibodies raised against mosquito midgut lysates lowered vector competence, reducing transmission of human malarial parasites [10], while antibodies to a sandfly midgut galectin eliminated sandfly transmissible infections of leishmania [11] in membrane feeding studies.

Arthropod vectors induce immunosuppression in the host during feeding and secrete pathogen transmission-enhancing factors that counteract host rejection responses [12–15]. For example, the Lyme disease agent Borrelia burgdorferi appears to exploit tick salivary proteins (BIP and Salp15 from I. ricinus and *I. scapularis,* respectively) to facilitate transmission to the mammalian host [16,17]. Therefore research on antivector immunity needs to be refined to distinguish antigens that induce protective immune responses whilst preventing pathogen transmission. Immunization with SP15 plasmid vaccine, targeting a 15-kDa saliva protein of the sand fly vector, *Phlebotomus papatasi,* induced humoral and delayed type hypersensitivity responses that successfully controlled Leishmania major infection in mice [18]. Immunity against the sand fly salivary antigen modified the dermal site of infection, limiting leishmania parasite replication although not apparently blocking transmission. Recombinant forms of a 15-kDa tick saliva protein (64TRPs) of Rhipicephalus appendiculatus likewise induce potent humoral and delayed type hypersensitivity responses [19]. In hamster, guinea pig, and rabbit models, this cement antigen acts as a dual-action vaccine by targeting the tick-feeding site (impairing attachment and feeding) and cross-reacting with “concealed” midgut antigens, resulting in death of engorged ticks [19,20]. The protein is derived from the cement cone that secures the tick's mouthparts in the host skin and, as a broad-spectrum vaccine antigen, is effective against adult and immature stages of several tick species, including I. ricinus [20]. In this study, we tested the potential of the 64TRP anti-tick vaccine to protect mice against a lethal infection of *tick-borne encephalitis virus* (TBEV) transmitted by its natural vector, *I. ricinus.* In humans, TBEV is the most important vector-borne virus infection in Europe and northern Asia [21]. Both transmission-blocking and protective activities were demonstrated by the 64TRP vaccine. Comparison of the results with those obtained when mice were immunized with either the commercial TBEV vaccine or the commercial anti-tick vaccine (TickGARD), together with histological and immunocytological studies, indicate that the key mode of action of 64TRP immunisation is the local cutaneous delayed type hypersensitivity response (possibly a CD8^+^ phenotype) evoked at the skin site of tick feeding.

## Results

### Effect of Immunizations on TBEV Transmission and Disease

**Figure 1:**
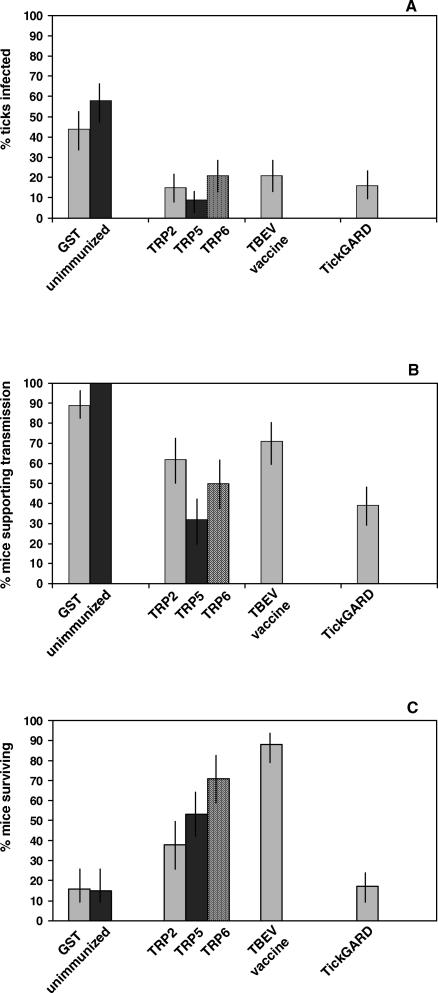
Effect of Immunization on Mice Infested with Virus-Infected and -Uninfected Ticks Comparison of immunization with either 64TRP antigens, a commercial TBEV vaccine, or the commercial anti-tick vaccine (TickGARD) on (A) transmission = % uninfected nymphal ticks that became infected; (B) support = % mice supporting cofeeding virus transmission between an infected adult female tick and uninfected nymphs; and (C) survival = % mice that survived an infected tick bite (only animals surviving subsequent i.p. inoculation with 1,000 PFU TBEV were included in the analyses).

Evaluation of the transmission-blocking and protective effects of immunization with either 64TRP tick cement antigens, TBEV virus, or TickGARD was based on comparing three parameters against controls: (1) “transmission”—virus transmission measured by the number of uninfected nymphal ticks that became infected when cofeeding with a single infected adult female; (2) “support”—number of mice supporting transmission from infected to uninfected ticks (this involves virus replication in mouse tissues) [22]; and (3) “survival”—number of mice that survived exposure to infected ticks (Figure 1). To ensure that surviving animals had been exposed to an infected tick bite, they were challenged by intraperitoneal (i.p.) inoculation of a lethal dose of TBEV. Animals that did not survive i.p. inoculation and did not yield infected ticks were excluded from the analyses (see Materials and Methods, “Data analysis”). Overall there was a correlation between “survival” and “support,” but not between “survival” and “transmission”: mice that supported virus transmission were significantly less likely to survive (r = −0.557, *p* = 0.02), whereas there was no correspondence between the % nymphs infected and the % mice that survived (r = −0.180, *p* = 0.488).

#### Controls.

All the unimmunized mice supported virus transmission, with each animal yielding at least three infected nymphs (Table 1). Hence TBEV had been transmitted from the infected female tick to the mouse on which it was feeding, and then transmitted from the infected mouse to uninfected cofeeding nymphs. Similar results were obtained with control mice immunized with glutathione S-transferase (GST). Most of the control mice died within 9–11 d of exposure to a TBEV-infected tick. There was no evidence of an anti-tick effect: the % nymphs fed on control animals compared with the treated groups were similar. However, the experiments were not designed to assess the effect of 64TRP immunization on tick feeding and tick survival, as demonstrated previously [19,20]. Thus nymphal ticks were removed prior to repletion and drop-off (for virus detection) instead of being assessed for feeding success and survival.

**Table 1:**
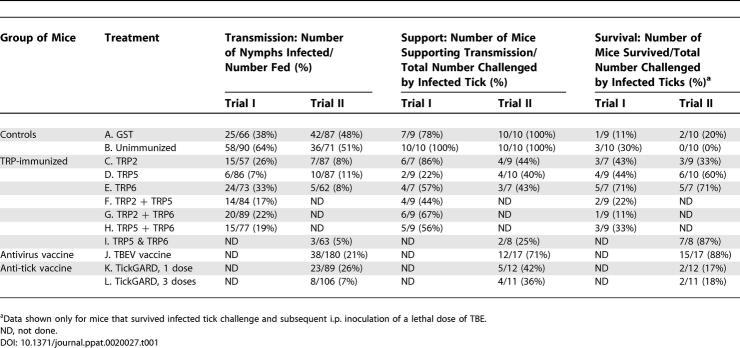
Evaluation of the 64TRP Anti-Tick Vaccine in Protecting Mice against Transmission of a Lethal TBEV Infection by Its Natural Vector, *I. ricinus,* and Comparison with Commercial Antiviral (FSME-IMMUN) and Anti-Tick (TickGARD) Vaccines

#### Effect of 64TRP immunizations.

Transmission-blocking activity was observed for 64TRP-immunized mice. Significantly fewer animals supported virus transmission to nymphs (χ^2^ = 39.2, df = 3, *p* ≤ 0.001) and fewer nymphs became infected (χ^2^ = 46.1, df = 1, *p* < 0.001) compared with the controls (Figure 1B and 1A, respectively). In addition, 64TRP immunization provided protection against a fatal infection with TBEV. Significantly more 64TRP-immunized mice survived compared with the controls (χ^2^ = 225, df = 1, *p* < 0.001) (Figure 1C). The highest level of protection from a single 64TRP dose was observed with TRP6 (Table 1). Immunization with the 64TRP cocktails, including two 64TRP doses (Table 2, group I), gave results that were not significantly different from the single immunogens (Table 1, group I).

**Table 2:**
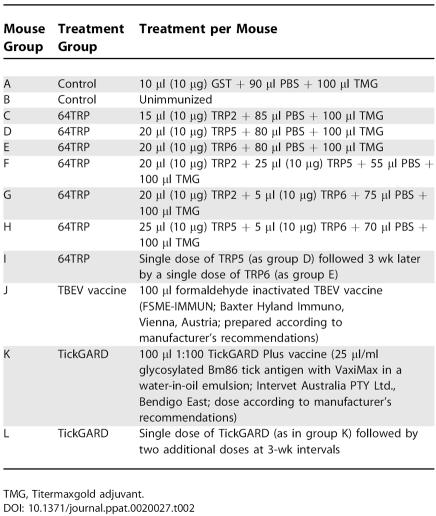
Immunization Regimes

#### Effect of TBEV vaccine.

The virus vaccine was similar to 64TRP in reducing the level of virus transmission to cofeeding ticks (Figure 1A). However, whereas significantly fewer 64TRP-immunized mice supported virus transmission compared with the controls, this was not the case for mice immunized with the virus vaccine (χ^2^ = 5.83, df = 7, *p* = 0.735) (Figure 1B). The protective effect of immunization with a single dose of the 64TRP tick antigens did not differ significantly from a single dose of the TBEV vaccine (χ^2^ = 5.83, df = 7, *p* < 0.560) (Figure 1C).

#### Effect of TickGARD vaccine.

Immunization with the commercial TickGARD vaccine (Table 2) provided transmission blocking similar to that observed following 64TRP-immunization (χ^2^ = 10.5, df = 7, *p* < 0.162). Compared with the controls, both TickGARD and 64TRP treatments gave lower transmission (χ^2^ = 32.1 df = 1, *p* = < 0.001), and fewer mice supported transmission (χ^2^ = 27.2, df = 2, *p* = < 0.001) (Figure 1A and 1B, respectively). However, there was a significant difference in protective effect of the tick cement–derived vaccine (64TRP) compared with the tick midgut vaccine (TickGARD) (χ^2^ = 52.2, df = 15, *p* < 0.001). Unlike 64TRP, TickGARD did not protect mice against a fatal infection with TBEV (Figure 1C), even when the animals were immunized with three doses of the commercial tick vaccine (Table 1, group L).

### Cellular and Humoral Immune Responses

A protective anti-tick immune response that blocks tick-borne pathogen transmission is associated with development of tick-specific antibodies [23,24] and cellular infiltration [25,26]. Therefore, we compared the responses of 64TRP-immunized mice with those of controls to identify a possible immunological basis for the observed transmission-blocking and protective effects. Differential humoral and cellular responses were observed in 64TRP-immunized mice compared with controls. Enzyme-linked immunosorbent assay results showed that antibody titers to cognate 64TRPs in 64TRP-immunized mice increased 2- to 4-fold relative to preinfestation titers (from a basal level of 1:4,000 to 1:8–16,000), whereas antibodies to GST had fallen in GST-immunized mice (to <1:1,000) after infestation. TBEV antibody titers were not determined.

**Figure 2:**
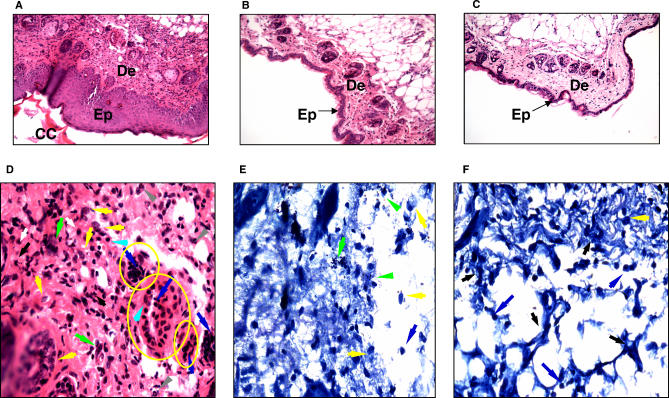
Skin Histological Response in Immunized Mice Infested with Virus-Infected and -Uninfected Ticks Histological profiles of skin sections taken at d 4 of TBEV-infected I. ricinus tick challenge on Balb/c mice immunized with either TRP2 (A, D), TRP5 (E), or GST (C, F), or unimmunized (B). Stained with hematoxylin and eosin (A–D) or “Hema Gurr” Rapid stain BDH (E, F) [19]. (A) (magnification 20×) TRP2-immunized animals, (B) (magnification 20×) control unimmunized, and (C) (magnification 20×) GST-immunized animals. Ep, epidermis; De, dermis; CC, cement cone of I. ricinus. (D–E) (magnification 63×) denote skin sections from TRP2 and TRP5 immunized mice, respectively, showing: perivascular cuffing = yellow circle; degranulating mast cells = green arrow; numerous lymphocytes = blue arrow; frequent macrophages = yellow arrow; and some eosinophils = white arrow; dermal dendrocytes = black arrow; neutrophils = gray arrow; and basophils = light blue arrow. (F) (magnification 63×) skin sections from GST control immunized mice showing lymphocytes, macrophages, and dermal dendrocytes.

In response to tick infestation, marked cellular infiltration was evident in skin sections of 64TRP-immunized animals. By contrast, comparatively little cellular response to tick feeding was observed in GST-immunized and unimmunized controls (Figure 2). For example, TRP2 immunization showed pronounced epidermal hyperplasia at the attachment site, with characteristic epidermal cavities and intense leukocytic infiltration in the dermis (Figure 2A), contrasting with the control unimmunized (Figure 2B) and GST immunized (Figure 2C) animals. The cement cone of I. ricinus showed some leukocytes present (Figure 2A). In addition, skin sections from TRP2 and TRP5 immunization revealed perivascular cuffing, degranulating mast cells, numerous lymphocytes, frequent macrophages, and some eosinophils, dermal dendrocytes, neutrophils, and basophils (Figure 2D and 2E). Fewer cells and cell types were observed in the GST control, and they were mainly lymphocytes, macrophages, and dermal dendrocytes (Figure 2F).

**Figure 3:**
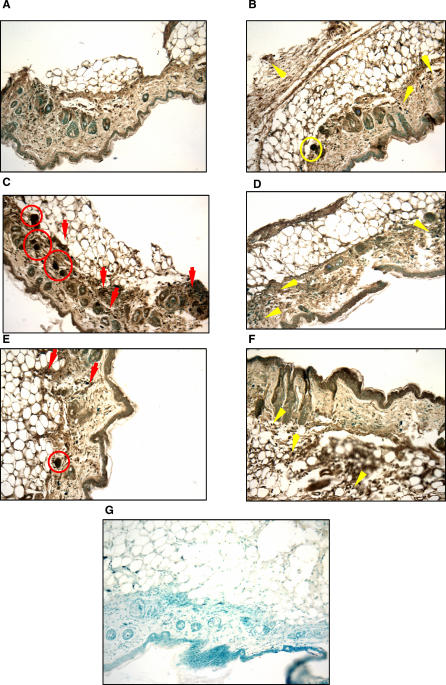
Skin Immunocytochemical Profile of Immunized Mice Infested with Virus-Infected and -Uninfected Ticks Immunocytochemical profiles of skin sections taken at d 4 of TBEV-infected I. ricinus tick challenge on Balb/c mice immunized with either GST (A, B) or TRP5 (C, D), or unimmunized (E, F), using rat anti-mouse CD4^+^ antiserum (B, D, and F) and rat anti-mouse CD8^+^ (A, C, and E) antiserum, with a negative control sample (G, PBS plus normal rabbit serum). (C) TRP5-immunized mice: red arrowheads = numerous CD8^+^ T cells; red circles = CD8^+^ T cells occluding the dermal blood vessels; and (D) yellow arrows = CD4^+^ T cells. (E) and (F) unimmunized mice, few CD8^+^ T cells = red arrows and CD4^+^ T cells = yellow arrows/yellow circle, respectively. (B) Control GST-immunized mice yellow arrows = few CD4^+^ T cells. (G) PBS-negative control skin sample = no T cells. Magnification 20×.

Evaluation of CD4^+^ and CD8^+^ T lymphocyte responses revealed a CD8^+^ response in 64TRP-immunized mice compared with GST and unimmunized animals (Figure 3). For example, skin samples from the TRP5-immunized mice showed a predominantly CD8^+^ T cell response, with numerous T cells occluding several dermal blood vessels (Figure 3C and 3D); a CD4^+^ T cell response was also observed but to a lesser degree (Figure 3D). In contrast, the primary response detected in the dermis of skin samples from control unimmunized mice following tick infestation was a CD4^+^ T cell response (Figure 3F) with a slight CD8^+^ effect. Control samples from GST-immunized mice were negative for CD8^+^ T cells (Figure 3A), with a slight CD4^+^ T cell response (Figure 3B). The characteristic epidermal hyperplasia observed in the histological profiles coincided with the site of tick attachment (Figure 2A, which shows the cement cone). Attachment sites were not observed in sections used for immunocytochemical staining, which may explain the apparent absence of an epidermal hyperplasia (Figure 3C and 3D).

## Discussion

In the mouse model for tick-borne transmission of TBEV, the virus is transmitted from an infected tick to the uninfected mouse on which it feeds. The virus infects and replicates in the skin site of feeding, and is then transmitted from the infected mouse to uninfected ticks cofeeding on the animal [23]. These transmission dynamics were observed for the control unimmunized and GST-immunized mice. Of the 39 control mice, 95% supported virus transmission giving rise to infected nymphs, and 51% uninfected nymphs feeding on the control mice became infected. Only 15% of the mice survived demonstrating the susceptibility of Balb/c mice to TBEV infection.

By comparison with the controls, transmission-blocking activity was observed in 64TRP-immunized mice. Only 48% animals supported virus transmission to nymphs and fewer nymphs became infected (16%) compared with the controls. Remarkably, an average of 46% 64TRP-immunized mice survived tick-transmitted virus infection and subsequent lethal challenge by virus inoculation. The highest level of protection from a single 64TRP dose was observed with TRP6 (71% survival). This construct shows the most extensive antigenic cross-reactivity with whole nymphal extracts, cement cone, and midgut of female I. ricinus [20]. 64TRP-immunized mice developed antiviral protection even when they did not support virus transmission to cofeeding nymphs. For example, of the ten surviving TRP5-immunized mice, eight animals did not yield infected nymphs, whereas all surviving control mice produced infected nymphs. These data indicate that the response of 64TRP-immunized mice to tick feeding did not completely block virus transmission but instead allowed sufficient exposure to the virus for the mouse to develop protective immunity. Thus virus transmission occurred from the infected female tick to the mouse, but not from the mouse to uninfected cofeeding nymphs. The experimental design did not allow us to distinguish immunization that completely blocked virus transmission from infected ticks to mice, leaving the mice still susceptible to infection. These animals would have been excluded from the analyses because they would not have survived the subsequent i.p. challenge with a lethal dose of TBEV.

The protective effect of immunization with a single dose of the 64TRP tick antigens did not differ significantly from a single shot of the commercially available inactivated TBEV vaccine (FSME-IMMUN; Baxter, Vienna, Austria) most commonly used in Europe [21]. The virus vaccine was also similar to the 64TRP vaccine in reducing the number of cofeeding ticks that acquired the infection. However, whereas significantly fewer 64TRP-immunized mice supported virus transmission compared with the controls, this was not the case for mice immunized with the virus vaccine. Thus, under the conditions used, the TBEV vaccine was not as effective as 64TRP in controlling transmission from the infected tick to the immunized mouse and/or from the infected mouse to uninfected cofeeding ticks. The results confirm a previous study in which 89% natural rodent hosts immune to TBEV supported cofeeding virus transmission [27], and indicate a greater level of virus infection in virus-immune mice compared with tick-immune mice. The contrasting results also indicate a different mechanism of protection, which is consistent with the lack of antigenic cross-reactivity between 64TRP antigens and TBEV proteins (unpublished data). The TBEV vaccine is presumably controlling the infection in the mouse, protecting the mouse against development of a fatal encephalitis and reducing the number of nymphs that become infected. By contrast, 64TRP appears to affect transmission from the infected tick to the mouse, and from the infected mouse to uninfected cofeeding nymphs, possibly acting at the level of Langerhans cells, which play a role in tick-borne TBEV transmission and are modulated by component(s) in tick saliva [22,28].

Immunization with the commercial TickGARD vaccine provided transmission-blocking similar to 64TRP-immunization, with both significantly lower transmission rates to nymphs and fewer mice supporting transmission compared with controls. However, unlike 64TRP-immunization, the transmission-blocking effects of TickGARD did not provide protection against lethal infection with TBEV. TickGARD and Gavac (the Cuban equivalent) are derived from Bm86, a midgut antigen of unknown function [29]. These commercial anti-tick vaccines induce an antibody-mediated response targeted at midgut cells that results in rupture of the midgut, tick mortality, and reduced reproductive output*.* They appear to reduce the incidence of tick-borne diseases in cattle (babesiosis and anaplasmosis) by depleting tick numbers [29]. The 64TRP antigen derived from a tick cement protein acts in a similar way to the Bm86 antigen: 64TRP cross-reacts with antigenic epitopes in the tick midgut, causing rupture to the midgut resulting in mortality [19,20]. Although our experimental design did not allow evaluation of the vaccination effects on tick feeding/survival, the transmission-blocking effects of TickGARD indicate that it affected the ability of nymphs to acquire the infection. This is consistent with the lack of correlation between the % nymphs infected and % survivors. A previous study using 64TRP-immunized rabbits and hamsters showed cross-protection against I. ricinus adults and nymphs, causing increased mortality [20]. Thus, anti-tick effects could increase the effectiveness of 64TRP as a protective transmission-blocking vaccine by reducing the tick vector population.

Mice immunized with 64TRP antigens reacted to tick infestation with both humoral and cellular responses. The increase in antibody titers in 64TRP-immunized mice relative to preinfestation titers (not observed in GST-immunized mice) indicates the secreted cement proteins of feeding ticks elicited an anamnestic response. The differential humoral and cellular responses of 64TRP-immunized mice compared with controls were similar to those observed in guinea pigs, rabbits, hamsters, and Boran cattle immunized with priming and booster doses of 64TRP compared with GST preparations ([19,20] and unpublished data). These consistent responses of 64TRP-immunized animals to tick feeding show that natural tick infestation stimulates anti-64TRP immunity, indicating the potential of a 64TRP-based vaccine to provide long-lasting immunity without the need for repeat vaccinations.

The humoral immune response observed is consistent with the histological profile of marked epidermal hyperplasia and infiltration of leukocytes following tick infestation of 64TRP-immunized animals contrasting with comparatively little cellular response to tick feeding in GST-immunized and unimmunized controls. Furthermore, T and B lymphocytes, plasma cells, macrophages, and dendritic cells are associated with acquired immunity [30]. Interestingly, the 64TRP-immunized mice demonstrated a predominantly CD8^+^ response compared with controls. Previous studies show that tick resistance is more effectively transferred with lymph node cells than with only serum from tick-resistant animals [31]. They imply an important role for cell-mediated immunity and the need to determine T cell phenotype. Our study is limited but the first to indicate the predominant phenotype of the T cell response invoked by the 64TRP tick cement antigen in a mouse model. A previous study reported CD4^+^ cells outnumbering CD8^+^ cells in mice repeatedly infested with I. ricinus nymphs that did not develop resistance to tick infestation, indicating that a predominant CD4^+^ cell type does not confer tick resistance in Balb/c mice [26]. Not surprisingly, therefore, repeat tick infestation does not induce full protection in laboratory mice [26]. By contrast, in other host species (guinea pigs, rabbits, and cattle), innate resistance to ticks is conserved during repeat tick infestations [32]. Moreover, humans repeatedly infested with I. ricinus ticks express dermal and perivascular infiltrates of CD8^+^ T lymphocytes [25]. Thus the predominant CD8^+^ profile in Balb/c mice immunized with 64TRP, and comparatively insignificant CD4^+^ response, are consistent with previous reports implicating the CD8^+^ phenotype in tick resistance.

Although it was not specifically determined, the observed antivirus immunity is unlikely to be CD8 dependent. Protection against lethal TBEV infection is not T cell mediated but has been linked to antibodies against a nonstructural virus protein absent from the virion [33]. Other studies in C57BL6 mice indicate that activation of virus-specific memory B cells to secrete IgG is independent of cognate or bystander T cell help [34]. Induction of complement is the dominant mechanism of protection, although when this pathway is inactivated other mechanisms involving cytotoxic T lymphocytes and natural killer cells come into play. T cells from immunized mice are protective only in animals whose pre-existing immune function had been impaired [35].

The marked cellular response to tick infestation in 64TRP-immunized mice, contrasting with controls, indicates that the immunomodulated site of tick feeding is disrupted. This disruption would also counter the activity of saliva-activated transmission factors present in I. ricinus saliva that promote TBEV transmission [36]. The TBEV saliva-activated transmission factor(s) has not been identified but appears to be secreted after the tick cement proteins; hence, it is unlikely to be the same molecule [27,37]. Thus, the observed protective effects in 64TRP-immunized mice most likely result from general humoral and cellular responses to tick feeding rather than specific antiviral or anti–saliva-activated transmission factor immunity. Our results indicate that TBEV transmitted by infected ticks into the skin of 64TRP-immunized mice enters a hostile microenvironment (e.g., Figure 2A) in contrast to the privileged feeding site in unimmunized mice (e.g., Figure 2B). According to the known dynamics of tick-borne TBEV infection in mice, disruption of the immunomodulated site of tick feeding prevents or limits establishment of TBEV at the initial skin infection site, a prerequisite of successful transmission [22]. This basis for the transmission-blocking and protective effects of 64TRP immunizations explains the contrasting observations with the TBEV and TickGARD vaccines since neither of these vaccines is reported to cause an inflammatory response following tick bite.

There is now convincing evidence that blood-feeding arthropod vectors are more than a mere syringe in transmitting vector-borne pathogens. The effect of their saliva at the skin site of feeding provides conditions that facilitate pathogen transmission and infection. Examples include sandflies and leishmania [38], ticks and tick-borne viruses and bacteria [37], mosquitoes and Cache Valley and La Crosse viruses [12,39], blackflies and vesicular stomatitis virus [14], mites and scrub typhus bacteria [40], mosquitoes and West Nile virus [41], and possibly *Aedes* mosquitoes and avian malaria parasites [42]. A vaccine strategy aimed at creating an antivector cell-mediated immune response at the skin site of transmission that interferes with pathogen transmission offers a new approach to controlling a vast range of important vector-borne diseases.

## Materials and Methods

### Immunizations.

Experimental Balb/c mice were arranged into treatment groups of at least 10 animals per group (Table 2). The following constructs of 64TRP, the tick cement protein, were used either singly or as cocktails: TRP5 (full-length soluble protein), TRP6 (full-length denatured protein), or TRP2 (a C-terminal truncation), expressed in Escherichia coli as GST-fusion proteins. The constructs were as described previously [19]. Controls were mice immunized with recombinant GST alone or naive animals that were not immunized. The 64TRP constructs and GST control were emulsified in Titermaxgold adjuvant (CytRx USA, Los Angeles, California, United States). In Trial I, 80 Balb/c mice were divided into 7 experimental groups and in Trial II, 62 mice were divided into five groups (Table 1). Mice were immunized with only a single priming dose (10 μg) of the 64TRP antigen (or GST control) unless otherwise specified (Table 2). All immunizations were by subcutaneous inoculation into the prescapular region.

### Serological assays.

Individual host immune responses to 64TRP immunizations were determined at wk 2 and 4, and at wk 6 after vaccination (i.e., pre- and postchallenge antibody titers, respectively) by end-point dilution enzyme-linked immunosorbent assays and strip blot assays using methods described previously [19].

### TBEV-infected ticks.

Ticks were obtained from a laboratory colony of uninfected I. ricinus maintained at the Institute of Virology, Bratislava, Slovakia. TBEV donor ticks were prepared by parenteral inoculation of I. ricinus females with 0.002 ml/tick TBEV (Hypr strain), diluted 10^−1^. Each tick received a dose of 5,000 plaque-forming units (PFUs) of virus. Each animal was infested with a single infected female tick accompanied by a single uninfected male tick to promote female tick feeding. Virus titration was as previously described [22]*.* After tick feeding, salivary glands were dissected from fed adult female ticks and assayed for TBEV; titers ranged from 100 to >1000 PFU. Nymphs that fed for 3 d (average feeding period) were tested for acquired TBEV; dead and unfed ticks were discarded.

### Host challenge with TBEV-infected tick.

When antiserum titers to the 64TRP constructs reached 1:4,000 to 1:16,000 (4 wk after immunization), each mouse was challenged with a single TBEV-infected female tick, cofed with one uninfected male and 15 uninfected nymphs in a transparent retaining chamber [22]. The same time interval (4 wk after immunization) was used to challenge the TBEV vaccine and TickGARD (Intervet Australia Pty. Ltd., Bendigo, Australia)–immunized mice. Mice that survived tick infestation were observed for clinical signs of encephalitis for a period of 21 d. After this observation period, to determine whether surviving mice had indeed been exposed to TBEV infection and developed protective immunity, each surviving animal was inoculated intraperitoneally (i.p.) with 1,000 PFU of TBEV (Hypr strain).

### Data analysis.

A Chi-squared test using binomial errors was performed to analyse the tick TBEV transmission efficiency data and analysis of variance with regression analysis to determine the correlations between mouse survival and transmission blocking/virus replication. All data from mice infested with uninfected ticks, or where ticks were not feeding or were destroyed by their host, were excluded from the analyses on the assumption that TBEV transmission had not occurred. Further, mice that did not support virus transmission between cofeeding ticks and did not develop protective immunity (they succumbed to i.p. inoculation of TBEV) were also excluded from the analyses. Although the conditions used to measure the level of protection were probably far more stringent than an infected tick bite, it was assumed that transmission and infection of the host had not occurred in survivors that were susceptible to i.p. TBEV challenge.

### Histological and immunocytochemical assays.

The nature of the cutaneous local inflammatory responses at tick feeding sites was determined by necropsy studies on skin biopsies after terminal anaesthesia, as previously described [19]. Wax-embedded skin sections were stained with hematoxylin and eosin or with Hema “Gurr” Rapid Blood Smear reagents. Phenotypes of T cell lymphocytes infiltrating the skin of 64TRP-immunized and control mice were characterized using antibodies to mouse cell-surface markers CD4^+^ and CD8^+^ by immunochemical staining using CD4^+^ or CD8^+^ antiserum (provided by Dunn School of Pathology, Oxford University, Oxford, United Kingdom).

## Supporting Information

### Accession Numbers

The GenBank (http://www.ncbi.nlm.nih.gov/Genbank) accession number for tick cement protein is AF469170.

## Abbreviations

GST: glutathione S-transferase
PFU: plaque-forming unit
TBEV: tick-borne encephalitis virus
